# Levan Enhances Associated Growth of Bacteroides, Escherichia, Streptococcus and Faecalibacterium in Fecal Microbiota

**DOI:** 10.1371/journal.pone.0144042

**Published:** 2015-12-02

**Authors:** Kaarel Adamberg, Katrin Tomson, Tiina Talve, Ksenia Pudova, Marju Puurand, Triinu Visnapuu, Tiina Alamäe, Signe Adamberg

**Affiliations:** 1 Department of Food Processing, Tallinn University of Technology, 19086 Tallinn, Estonia; 2 Competence Center of Food and Fermentation Technologies, 12618 Tallinn, Estonia; 3 Department of Genetics, Institute of Molecular and Cell Biology, University of Tartu, Riia 23, 51010 Tartu, Estonia; 4 Department of Systems Biology, Technical University of Denmark, Elektrovej, building 375, 2800 Kgs. Lyngby, Denmark; Indian Institute of Science, INDIA

## Abstract

The role of dietary fiber in supporting healthy gut microbiota and overall well-being of the host has been revealed in several studies. Here, we show the effect of a bacterial polyfructan levan on the growth dynamics and metabolism of fecal microbiota *in vitro* by using isothermal microcalorimetry. Eleven fecal samples from healthy donors were incubated in phosphate-buffered defined medium with or without levan supplementation and varying presence of amino acids. The generation of heat, changes in pH and microbiota composition, concentrations of produced and consumed metabolites during the growth were determined. The composition of fecal microbiota and profile of metabolites changed in response to substrate (levan and amino acids) availability. The main products of levan metabolism were acetic, lactic, butyric, propionic and succinic acids and carbon dioxide. Associated growth of levan-degrading (e.g. *Bacteroides*) and butyric acid-producing (e.g. *Faecalibacterium*) taxa was observed in levan-supplemented media. The study shows that the capacity of levan and possibly also other dietary fibers/prebiotics to modulate the composition and function of colon microbiota can be predicted by using isothermal microcalorimetry of fecal samples linked to metabolite and consortia analyses.

## Introduction

The breakdown of complex carbohydrates is one of the most important processes carried out by colon microbiota that plays a central role in biochemical and physiological processes not only in the colon, but also in other places of the body. Fermentation processes in the colon are regulated, in large part, through substrate availability [1]. Microbially accessible carbohydrates (MACs) are carbohydrates that are metabolically available to gut microbes. The MACs include dietary fibers (food components which are resistant to degradation and absorption in the upper digestive tract of the host), carbohydrate components of intestinal mucus and sugars produced by microbes within the intestine. Dietary fibers originate from a variety of sources including plants, animal tissues and food-borne microbes. The amount of carbohydrates metabolized depends upon composition of the person’s microbiota and differs largely between individuals [2].

Diet is among the easiest way to manipulate the composition and activity of gut microbiota [3–6]. However, little is known about how these changes can be correctly predicted [3]. The microbial fermentation of complex dietary components in the colon occurs in trophic chains and insight into this multispecies-aided conversion is essential to understand the impact of the diet on health [7]. Co-inhabiting groups can efficiently use limited resources through metabolite sharing and exchange, thus providing a survival advantage and enabling coexistence of different bacterial species in diverse niches [8]. Fermentation of complex carbohydrates by colon microbiota results in production of organic acids, especially acetate, propionate and butyrate that fuel the enterocytes but also have—signaling functions for systemic immune and metabolic responses [7,9,10].

The effect of inulin (β-2,1-D-fructan) on dynamics and metabolism of fecal microbiota has been studied extensively *in vitro* and *in vivo* [10–12]. Another group of fructose polymers, levans (β-2,6-D-fructans), synthesized mostly by microorganisms but also by some plants are naturally present in various food products and consumed by humans in low quantities [13]. Microbial levans are more advantageous, economically and industrially feasible compounds with numerous applications. Levan is currently produced by many companies around the world and it is used in foods, beverages, medicine and nanotechnology [14,15]. However, levan is currently not in common use in Europe. With excellent biocompatibility and ease of production, microbial levan appears as a valuable and versatile biopolymer of the future [14]. We consider that levan should act as a selective growth substrate for gastrointestinal microbiota since β-2,6 glycosidic bonds characteristic for levan can be cleaved by only few bacterial species. For example, from tested *Bacteroides* species, only strains of *B*. *thetaiotaomicron* are able to grow on levan [16].

Many bacterial species including *Zymomonas mobilis*, *Bacillus subtilis* and *Lactobacillus reuteri* harbor levansucrase and synthesize levan from sucrose. Levan used in the current study was prepared using levansucrase Lsc3 of *Pseudomonas syringae* pv. tomato DC3000 [13] as described by Adamberg *et al*. [17] and subsequently dialyzed to remove residual amount of mono- and disaccharides.

The growth dynamics and metabolism of microbial consortia can be studied by using various cultivation techniques from simple batch to advanced continuous fermentations [18], whilst simpler methods with higher throughput are preferred for screening purposes. Due to the relative simplicity and cost-effectiveness the use of isothermal microcalorimetry (IMC) in biological and biomedical fields such as detection and characterization of pathogens, drug testing, parasitology and tissue engineering has been increasing over the last ten years [19]. The production rate of heat that reflects the cellular metabolic activities is measured on-line in case of IMC [20,21]. Sensitivity of this method compares well with commonly used techniques, and given proper data analysis, calorimetric data can be translated into meaningful biological equivalents (for example length of lag phase, growth and substrate consumption rate) [21]. IMC has proven especially useful, when dealing with opaque, solid, semi-solid or encapsulated samples [22]. Mathematical comparison of the power-time curves can allow identification of microorganisms or can simply be used for quality control purposes (i.e., a measure of the variation of a specific process monitored using its associated thermal power curve) [21].

The aim of the current study was to elucidate the modulatory effect of levan on the composition and dynamics of consortia of fecal bacteria using a novel approach on this field: microcalorimetry linked to metabolite profiling and microbiome characterization.

## Materials and Methods

### Fecal samples and levan

Eleven fecal samples were donated by healthy Estonian adult subjects (mean age 32 ± 12 years).

The fecal samples were collected into sterile 25 ml feces sampling containers (APTACA, Italy) and kept under anaerobic environment in a gas generating pouch system (GasPak™, Becton Dickinson & Co, USA) at -20°C. Frozen samples were delivered to the laboratory within 48 h and stored at -80°C until processing (no longer than three weeks). The storage cultures were prepared aseptically in a glove box flushed with nitrogen gas. The stool sample was diluted five times with sterile anaerobic PBS (mM): NaCl (160), KCl (3), Na_2_HPO_4_ (8), NaH_2_PO_4_ (1), containing 5% dimethylsulfoxide (DMSO) (vol/vol), pH 7.2, supplemented with freshly made and filter sterilized Cys-HCl (0.05% in final solution). The diluted samples were stored at -80°C as aliquots (5 ml) sufficient to start a cultivation experiment.

Levan was produced from sucrose using Lsc3 from *P*. *syringae* DC3000 and precipitated with ethanol as shown previously in Adamberg *et al*. [17]. To remove low molecular weight compounds including mono- and disaccharides, levan was dissolved in MQ water and dialyzed for 36 h at room temperature in membrane tubing of 20 kDa molecular weight cut-off against sterile milliQ water. The dialyzed levan preparation was freeze-dried (VirTis freeze dryer, SP Industries Inc., Warminster, USA).

### Isothermal microcalorimetry (IMC)

The growth experiments were performed as described in Adamberg *et al*. with some modifications [17]. The base medium for growth experiments contained: 0.05 M potassium phosphate buffer made from 1 M stock solutions (ml/L): K_2_HPO_4_ (35.85) and KH_2_PO_4_ (14.15) and mineral salts (mg/L): MgSO_4_*7H_2_O (36), FeSO_4_*7H_2_O (0.1), CaCl_2_ (9), MnSO_4_*H_2_O (3), ZnSO_4_*7H_2_O (1), CoSO_4_*7H_2_O (1), CuSO_4_*5H_2_O (1), MgCl_2_ (2), (NH_4_)_6_Mo_7_O_24_*4H_2_O (1), NaCl (527), (NH_4_)Cl (400); without and with levan (5 g/L) as a carbon source; pH 7.2.

The M20 medium contained additional amino acids and vitamins and it was supplemented with levan (5 g/L) [15]. Before inoculation, the growth media were prereduced in an anaerobic jar (Anaero-Gen™, Oxoid Inc.,UK), supplemented with stock solutions of freshly prepared and filter-sterilized Cys-HCl (concentration in the medium 0.5 g/L) and autoclaved sodium thioglycolate (concentration in the medium 0.5 g/L) as reducing agents. The media were inoculated with aliquots prepared from frozen feces to obtain approximately 10–100 times dilution.

The ampoules with total volume of 3.3 ml were filled with 2 ml of inoculated medium, closed hermetically and incubated at 37°C in a 24-channel isothermal microcalorimeter TAM III (TA Instruments, Delaware, USA) as described by Kabanova *et al*. [23]. The heat flow (P, μW) was recorded and total accumulated heat (Q, J) which is proportional to generated biomass was calculated by integration of the heat flow. All fecal samples were tested at least in two independent calorimetry experiments with two technical replicates in each experiment.

### Determination of metabolites

At the end of 48 h-incubation, the gas sample was taken for the composition analysis before opening the ampoule. Gas composition (H_2_, CO_2_, H_2_S, CH_4_, and N_2_) of the head space of the ampoules was analyzed using the Agilent 490 Micro GC Biogas Analyzer (Agilent 269 Technologies Ltd., USA) coupled with thermal conductivity detector as described in Adamberg *et al*. [17].

Samples from the beginning and end of the growth experiments were analyzed for microbial 16S rDNA sequences, metabolites and pH. The samples were centrifuged (21000 g, 10 min), pellet and supernatant were separated and stored at -20°C until the analysis. 10% sulfosalicylic acid (1:0.25; vol/vol) was added to the supernatant before freezing. Bacterial DNA was analyzed from cell pellets.

Before the chromatographic analysis, the samples were centrifuged (21000 g, 15 min, 4°C) and the supernatants were filtered through 0.20 μm PTFE syringe filters (Millex filters SLLGH13NK, Millipore, USA). The samples of fecal homogenate with high polysaccharide and/ or levan content were additionally ultra-filtered using AmiconR Ultra-10K Centrifugal Filter Devices, cut-off 10 kDa (Millipore, USA). The concentrations of organic acids (succinate, lactate, formate, acetate, propionate, isobutyrate, butyrate, isovalerate, valerate), glycerol and ethanol were determined by high-performance liquid chromatography (HPLC, Alliance 2795 system, Waters, Milford, MA, USA), using a BioRad HPX-87H column (Hercules, CA, USA) with isocratic elution of 0.005 M H_2_SO_4_ at a flow rate of 0.6 mL/min and at 35°C. Refractive index (RI) (model 2414; Waters, USA) and UV (210 nm; model 2487; Waters, USA) detectors were used for quantification of the substances. Detection limit for the HPLC method was 0.1 mM.

Concentrations of amino acids and amines were determined using pre-column derivatization of amino acids and amines by 6-aminoquinolyl-N-hydroxysuccinimidyl carbamate and UPLC (Waters, Milford, USA) according to the manufacturer’s instructions. Empower software (Waters, USA) was used for the processing of HPLC and UPLC data. Detection limit of the UPLC was 0.01 mM.

Total content of levan in the supernatants was determined by anthrone assay as described in Adamberg *et al*. [17]. Standards containing 5–100 mg/L fructose and 0.6 M HCl (final concentration) in deionized water were analyzed with each run. To account for the absorbance due to stool components, a second blank was introduced made by adding sulphuric acid reagent without anthrone, when the final dilution of feces in the sample was 1:100 or less. All measurements were performed in triplicate.

### DNA extraction and amplification

DNA was extracted from all samples using MoBio PowerFecal DNA extraction kit (MoBio, Carlsbad, CA, USA) according to the manufacturer’s instructions. Universal primers:

S-D-Bact-0341-b-S-17 Forward 5´ TCGTCGGCAGCGTCAGATGTGTATAAGAGACAGCCTACGGGNGGCWGCAG F and

S-D-Bact-0785-a-A-21 Reverse 5´ GTCTCGTGGGCTCGGAGATGTGTATAAGAGACAGGACTACHVGGGTATCTAATCC were used for PCR amplification of the V3-V4 hypervariable regions of the 16S rRNA genes [24]. The amplified region was about 450 bp and on average 12000 reads per sample were obtained. The mixture of amplicons was pyrosequenced using Illumina MiSeq 2x250 v2 platform (Microsynth AG, Switzerland).

### Taxonomic profiling of fecal samples

The sequencing data were analyzed using the program MOTHUR v1.35.0 according to the instructions of standard operating procedure [25,26]. PCR errors and reads shorter than 400 bp or containing more than eight homopolymers were removed from the dataset. Sequences were aligned to the SILVA reference 16S rRNA database and clustered into Operational Taxonomic Units (OTUs) according to average neighbor clustering algorithm based on 97% sequence identity. Finally, the dataset of each sample was normalized according to the sample with lowest quantity of sequences. The taxonomy of each OTU was assigned using SILVA 16S rRNA database (v119) (or the Greengenes database v13.5).

### Statistical analysis

For multivariate analysis, data of all experiments (abundances of bacterial taxa, growth characteristics from calorimetry and metabolites’ production/consumption) were merged into a matrix table. Partial least squares discriminant analysis (PLS-DA) of the data was performed using web-based software MetaboAnalyst 3.0 [27].

### Ethics statement

During a preparatory consultation interview, the volunteers were informed about the scope of research and all the subjects gave written informed consent. The study was approved by the Tallinn Medical Research Ethics Committee, Estonia (protocol No. 554).

## Results

### Growth dynamics based on power-time curves and 16S analysis of the bacterial consortia

**Fig 1:**
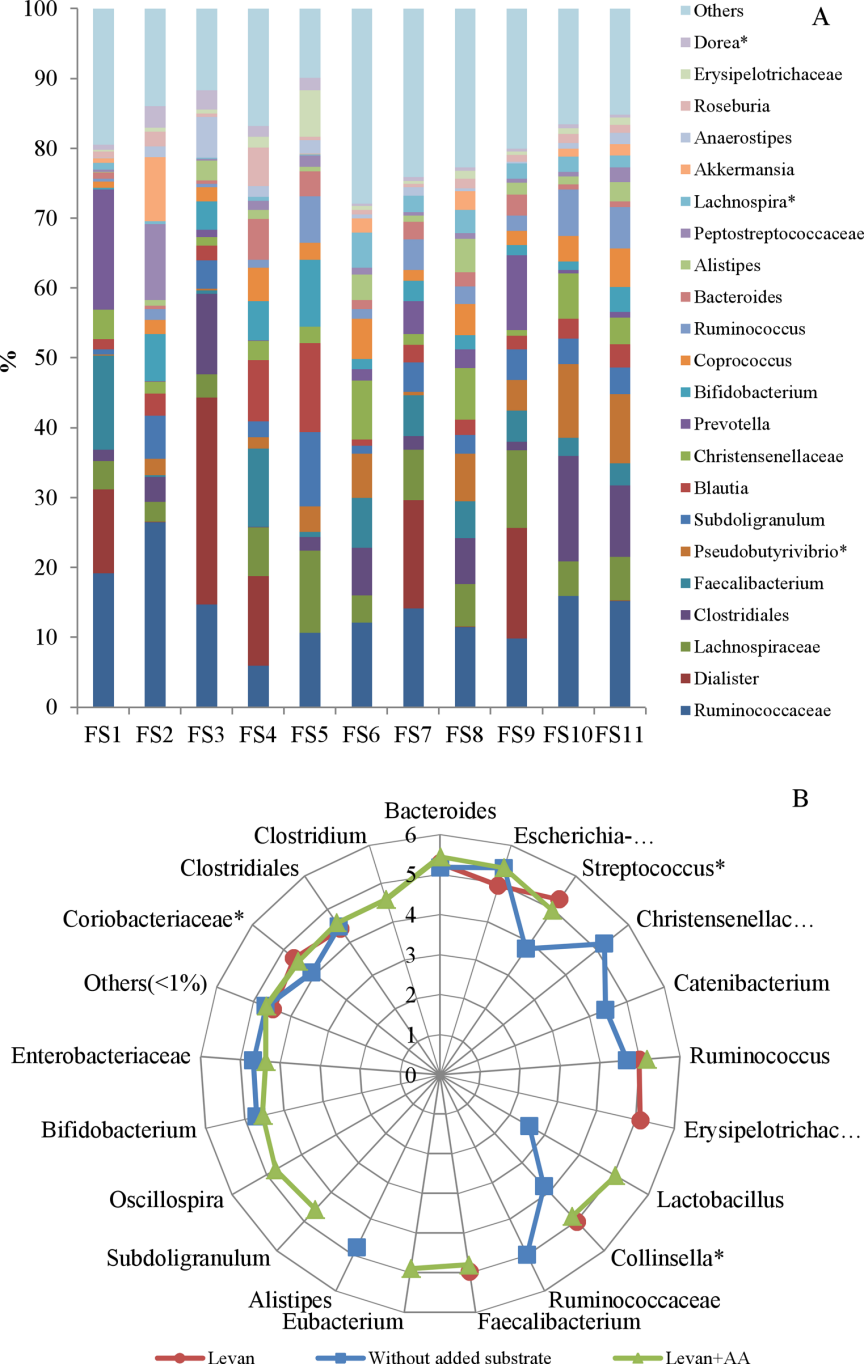
Bacterial taxa in individual fecal samples and their abundance after the growth in microcalorimeter. (A) 22 bacterial taxa of fecal samples (FS1-11) with average abundance ≥ 1% are shown (sum of reads in relative scale, %). (B) abundance of genera (family/order level if the genus was not identified) per million cells (1 read = 1 cell) after growth on levan (red dots), levan plus amino acids (green triangles) or without added substrates (blue rectangles) in a logarithmic scale. All data points presented as average values of all fecal samples. *—statistical difference between the samples with levan and without added substrates (p-value < 0.05), **—statistical difference between the samples with levan and levan + amino acids (p-value < 0.05).

In total, 325 operational taxonomic units (OTU-s) with a relative abundance over 0.1% were found in at least one out of 11 fecal samples. Initial compositions of all eleven fecal consortia (FS1–11) were dominated (abundance over 50%) by species from the phylum *Firmicutes*. The major taxa represented in the samples belonged to *Ruminococcaceae*, *Lachnospiraceae*, *Dialister*, *Faecalibacterium*, *Pseudobutyrivibrio*, *Subdoligranulum*, *Blautia*, *Prevotella*, *Bifidobacterium*, *Coprococcus* (Fig 1A, S1 Table). Interestingly, the genus *Dialister* was dominating in five fecal samples (FS1, FS3, FS4, FS7 and FS9), yet no enrichment of this taxon was observed during cultivation with levan or levan plus amino acids. According to the literature, *Dialister invisus* has been associated with human clinical samples and infections [28]. Recently *Dialister*, *Roseburia* and *Bifidobacterium* genera were shown to be enriched among fecal microbiota by diet including whole-grain barley [29].

The growth experiments using fecal inocula were performed in defined media containing either 20 amino acids or only Cys with or without levan. The heat generation (biomass growth) of control cultures (base medium without levan and additional amino acids) depended on the amount of residual substrates (complex carbohydrates and proteins) in the fecal material accessible to microorganisms during the incubation. Taking into account the theoretical energy gain from carbohydrate fermentation in anaerobic environment and content of 20 amino acids added to the medium (in total 0.86 g/L), at least 2 g/L of fermentable substrates should be present in 10% fecal slurry (corresponds to approximately 10% of the initial fecal dry mass if dry matter in feces is 25% [30]).

**Fig 2:**
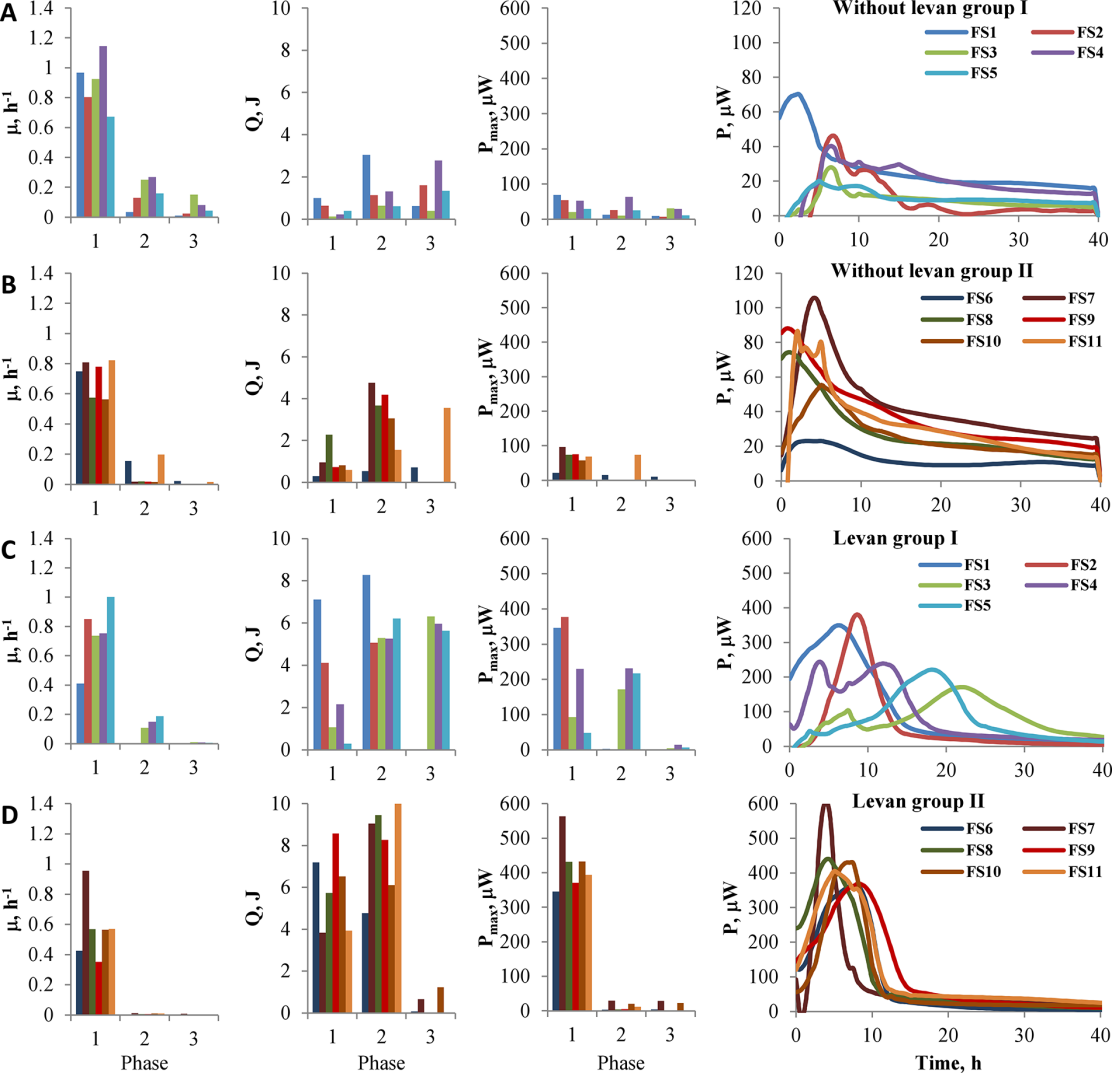
Growth characteristics of fecal consortia on media of varied composition. (A) Growth of fecal cultures from group I (FS1-5) on medium without levan and amino acids. (B) Growth of fecal cultures from group II (FS6-11) on medium without levan and amino acids. (C) Growth of fecal cultures from group I on medium with levan. (D) Growth of fecal cultures from group II on medium with levan. μ - specific growth rate (h^-1^), Q—heat accumulated (J) and P_max_—maximum heat evolution rates (power, μW) achieved in different growth phases. Growth phase is defined as time between the power peak maxima, *i*.*e*. each power peak divides growth into two phases: before and after power peak maximum. Error bars indicate standard deviation of 2–4 biological replicates. Line graphs illustrate the heat evolution rate (P–power, μW) during the whole experiment. Note that line graphs on consortia growth with and without levan have different Y axis scale.

Addition of 5 g/L of levan to basic medium significantly increased the growth of fecal biomass (up to 0.47 gDW/L; DW, dry weight) when compared to cultures without any carbon source added (up to 0.23 gDW/L, Fig 2). The main taxa enriched in levan-supplemented cultures comprised *Bacteroides*, *Streptococcus*, *Ruminococcus*, *Erysipelotrichaceae*, *Collinsella*, *Faecalibacterium* and *Coriobacteriaceae*. In addition, supplementation of levan + 20 amino acids supported growth of *Lactobacillus*, *Eubacterium*, *Subdoligranum*, *Oscillospira* and *Clostridium*. *Bifidobacteria* and *Enterobacteriaceae* were competitive in cultures with levan + amino acids, but remained in minority when grown on levan only, whilst *Erysipelotrichaceae* was not competitive in cultures with levan + amino acids (Fig 1B).

The fecal samples studied here were divided into two subgroups according to the obtained calorimetric curves exhibiting either a) multiauxic growth or b) growth with single major power peak in levan-supplemented media as well as in media without any carbon source added (Fig 2, complete data in S2 Table). The multiauxic growth on levan started with a small power peak (less than 100 μW) followed by prolonged major growth phase (samples FS1 and FS3-5). In the second group, initial growth on levan was fast with high heat evolution rate (P_max_ up to 600 μW), and no distinct growth were further observed. The growth curves in medium containing levan and twenty amino acids were similar to those with levan only (S2 Table, not shown in Fig 2). When compared with levan-supplemented media, the power-time curves on residual fecal substrates (without any substrates added) were more diverse displaying either a) several distinct growth phases (samples FS1-5), or b) only one plateau-like phase (samples FS6-10) with only small first power peak and total heat accumulation being significantly smaller (Fig 2A and 2B). The multiauxic heat evolution suggests that the excreted fecal material contains several types of substrates (mostly undigested carbohydrates and proteins) and reflects the flexibility of microbial consortia to switch from utilization of one substrate to another. In case of fecal cultures without added substrates, overgrowth of presumably proteolytic species from genera *Escherichia/Shigella* was observed. The total produced heat (sum of heat produced during different phases, Fig 2C and 2D) at growth in levan-containing medium varied about two times between different fecal samples (7.9±1.1 to 17.8±0.5 J) whilst much larger variation between the samples was observed if the consortia were growing on residual substrates only (1.6±0.9 to 5.7±2.1 J). This sample-specific feature possibly characterizes the energetic potential of the individual’s diet and/or efficiency of the colon microbiota to extract metabolic energy from undigested food components reaching the colon.

### Metabolism of carbohydrates and amino acids by fecal consortia

The main fatty acid found in fecal samples donated was acetate (3.1±1.1 mmol/gDW as average for 11 samples) followed by propionate and butyrate (0.6±0.5 and 0.5±0.4 mmol/gDW, respectively, Table 1). Minor amounts of succinate and isobutyrate (below 0.1 mmol/gDW) were also detected. Molar ratios 50:7:9 of acetate, propionate and butyrate in samples analyzed in this study were comparable to 42:16:14 ratios presented in Cummings *et al*. [31]. Fecal samples contained also small amounts of free amino acids, ranging from 0.002±0.002 (Trp) to 0.14±0.08 (Glu) mmol/g, γ-aminobutyric acid (GABA) and biogenic amines (see S3 Table).

**Table 1:** Content of organic acids in fecal cultures (μmol/g±SD) analyzed before each cultivation experiment (2–4 calorimetry replicates per sample). Complete data shown in the S3 Table.

Culture	Acetate	Butyrate	Isobutyrate	Propionate	Succinate
FS1	51.1 ± 3.2	10.7 ± 1.3	0.7 ± 1.0	14.4 ± 0.7	1.0 ± 0.4
FS2	25.5 ± 3.5	3.8 ± 1.8	2.0 ± 3.0	4.2 ± 2.2	1.5 ± 0.7
FS3	19.3 ± 2.4	3.8 ± 0.8	1.5 ± 2.2	2.2 ± 1.9	0.5 ± 0.5
FS4	46.2 ± 6.8	9.2 ± 1.2	2.9 ± 4.3	8.5 ± 1.9	2.4 ± 0.9
FS5	24.5 ± 1.0	4.3 ± 0.5	0.8 ± 1.3	5.8 ± 1.2	1.4 ± 0.5
FS6	23.3 ± 5.2	1.2 ± 0	0 ± 0	2.3 ± 2.3	0.3 ± 0.3
FS7	49.4 ± 0.8	9.5 ± 0	0.5 ± 0.5	16.3 ± 1.1	4.9 ± 0
FS8	27.7 ± 5.1	2.7 ± 0	1.0 ± 1.0	3.5 ± 2.2	0.3 ± 0.3
FS9	23.3 ± 2.4	1.5 ± 0	0.4 ± 0.4	4.8 ± 2.1	2.9 ± 0.3
FS10	25.5 ± 5.4	5.0 ± 0	0.1 ± 0.1	3.7 ± 2.7	0.3 ± 0.3
FS11	24.9 ± 0.2	1.2 ± 0	0 ± 0	2.8 ± 1.4	0.7 ± 0.2

**Fig 3:**
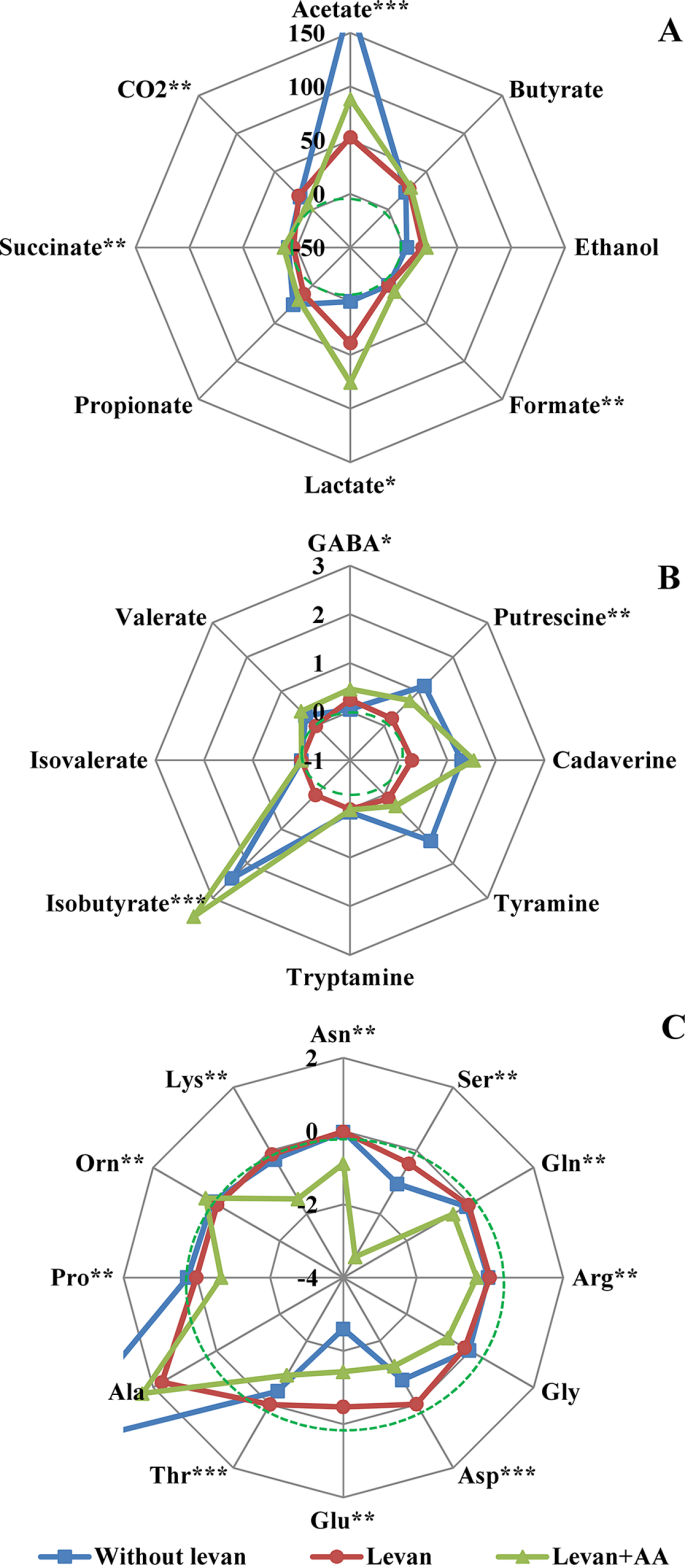
Production of organic acids, carbon dioxide and ethanol (A), biogenic amines and branched-chain fatty acids (B), and production/consumption of amino acids (C) (mmol/gDW) during growth of fecal consortia in media containing levan (in red), levan + amino acids (in green) or without added substrates (in blue). The data presents the average of all fecal samples (n = 11 with 1–4 biological replicates). Dotted line indicates the zero level (no production or consumption). *—significant difference between levan and no-substrate conditions (p-value < 0.05), **—significant difference between levan and levan + amino acids (p-value < 0.05), ***—significant difference between levan and levan + amino acids and no substrate conditions (p-value < 0.05).

Although the heat evolution curves at growth of fecal samples did not remarkably differ between the media containing levan or levan + amino acids, consumption or production profiles of metabolites differed significantly (Fig 3). During the incubation levan was completely consumed when considering the sum of formed products and only trace amounts of fructose (levan degradation product) were detected by anthrone assay (data not shown). At growth of fecal consortia on levan, mainly acetic and lactic acids (76±12 and 63±19 mmol/gDW, respectively, average confidence interval ±95%, Fig 3A) and about 2–3 times smaller amounts of butyric and propionic acids were formed. This result indicates simultaneous growth of bacteria producing those acids such as streptococci, *Escherichia*, *Bacteroides*, *Collinsella*, *Faecalibacterium*, *Eubacterium* and *Catenibacterium* (Fig 1B). To give a comparison, the molar ratios of acetate:propionate:butyrate were about 72:19:8 in case of growth of fecal batch cultures on inulin, and 58:18:25 on starch as reported by Wang and Gibson [11]. In our study equimolar production of acetic and lactic acids refers to the possibility that lactate was not completely fermented to butyrate or propionate, most probably due to accumulation of acids and decrease of pH. The level of acidity is a crucial factor for determination of growth and metabolism of gut microbiota [32,33]. The pH of the control cultures without additional substrates used was decreased at most by 1.5 units (from 7.1 to 5.6), whereas in levan-supplemented cultures it decreased to 4.7–5.2 (data not shown).

Small amounts of detected CO_2_ may indicate that anabolic routes from phosphoenolpyruvate to oxaloacetate or reductive TCA cycle were active, and thus intermediates of the TCA cycle were used for the biosynthesis or exported as unidentified compounds. Indeed, in accordance with this, we could not identify some peaks in HPLC chromatograms, and based on the model calculations the total carbon shortage was on average 15 C-mmol/L (difference between the total carbon consumed and total carbon produced in case of growth at levan supplementation was 150 C-mmol/L).

Amino acid synthesis should also be considered, because they were neither remarkably consumed nor produced in cultures in the presence of levan. Additional amino acid supply significantly affected central metabolism as well as amino acid metabolism (Fig 3). Degradation of amino acids to isobutyrate and biogenic amines was more intensive at growth in levan-containing medium supplemented with 20 amino acids compared to that in levan-containing medium with only Cys added (Fig 3B). Active amino acid degradation was accompanied with increased production of acetate, succinate and formate. The amino acids with highest consumption rates in M20 medium were Ser, Glu, Asp and Lys. The only amino acid produced in significant amounts in all experimental conditions was Ala (1.7, 2.3 and 5.3 mmol/gDW in media with levan, levan + 20 amino acids and without the added carbon source, respectively). Addition of amino acids also affected the dynamics of bacterial population by enhancing the growth of *Lactobacillus*, *Eubacterium* and *Subdoligranulum*, which was not observed in the medium containing only levan (Fig 1B).

During incubation of fecal slurries with neither levan nor amino acids added, mainly acetate was produced (177±81 mmol/gDW), while propionate and butyrate were produced in minor amounts (25±12 and 23±10 mmol/gDW, respectively, Fig 3A). Glu and Ser were significantly consumed whereas Ala was produced when grown on levan + amino acids (Fig 3C). Notably, biogenic amines were produced at significantly larger amounts during growth of fecal consortia without levan (Fig 3B) suggesting proteolysis of residual proteins present in fecal slurry as consumption of Lys, Arg, Tyr and Trp was not observed (Fig 3C, S4 Table). In the medium without added levan Ile, Val and Leu were also produced (S4 Table), which might be the result of hydrolysis of the proteins. Alternatively, synthesis of these amino acids may be promoted for reoxidation of coenzymes as in the case of Ala production in anaerobic bacterial fermentations and growth of *Aspergillus nidulans* in hypoxic conditions [34]. In levan-supplemented medium the fecal bacteria most likely use other possibilities to maintain the redox balance. The metabolites detected at growth in the absence of levan refer to domination of proteolytic bacteria such as *Enterobacteriaceae*, and suppression of butyric acid producing bacteria such as *Faecalibacterium* and *Ruminococcus*. Specific groups of bacteria detected only in medium without the addition of levan and amino acids were *Christensenellaceae*, *Catenibacterium* and *Alistipes*.

## Discussion

### Levan-induced changes in growth dynamics, metabolism and bacterial composition of fecal samples

The effect of β-2,1-linked fructans, inulin and FOS (fructooligosaccharides), on gut microbiota has been thoroughly studied [[10–12]]. Here we present the results on how a β-2,6-linked fructan levan and varied amino acid supplementation shapes the composition and metabolism of fecal microbiota. Our data obtained here support the data and opinion of other researchers [35,36] showing that degradation and metabolism of carbohydrates in complex microbial ecosystems proceeds through cross-feeding, and that the profile of end products is determined not only by the composition and structure of the degradable substrates, but also by composition of the consortium.

Addition of levan clearly promoted growth of fecal microbiota (Fig 2C and 2D). Two distinct fermentation subtypes were observed during growth of fecal cultures in levan-containing medium. In the case of the first subtype (Type I, samples FS1-5), growth proceeded through multiple phases: the fast minor growth phase was followed by several prolonged growth phases with highest heat generation in the last phase. The heat evolution rate in all growth phases was small and did not exceed 400 μW. The second subtype (Type II, samples FS6-11) can be characterized by fast growth within the first 20 hours suggesting efficient adaptation of these microbial consortia to assimilation of levan. Only a few growth phases were detected and most of the heat accumulated during the first and second phases had high heat evolution rates (up to 600 W, Fig 2D). The initial bacterial composition of fecal subgroups I and II was rather similar: from 22 dominant genera only the amounts of *Pseudobutyrivibrio*, *Lachnospira* and *Dorea* were significantly different between the subgroups (Fig 1A, S1 Table). Considering that the proportion of those bacterial groups did not increase during the fermentation either with or without levan, we propose that the heat evolution was obviously not influenced by these genera. The main genera enriched in levan-containing medium were *Bacteroides*, *Escherichia*, *Streptococcus* and *Ruminococcus*. Importantly, several taxa that are potential butyrate-producing bacteria such as *Erysipelotrichaceae* and *Faecalibacterium* were also enriched at levan supplementation (Fig 1B).

The power-time curves at growth of fecal samples without added substrates (at the expense of residual nutrient sources) reflect the amount of undigested components escaping human digestive enzymes and thereby become accessible to colon microbes. Therefore, minimal heat flow values of the fecal culture may refer to low consumption of dietary fiber by the fecal donor, or on the other hand, high activity of colon microbes on fiber substrates consumed by the resulting lower residual energy of feces. The experiments on gnotobiotic mice have indicated the increase of intestinal energy extraction and concordantly reduced feces energy level obtained from inulin compared to cellulose [12]. It should be elucidated in further experiments if there is a correlation between residual energy in feces, composition and activity of the colon microbiota and consumption of dietary fibers by the donor.

**Fig 4:**
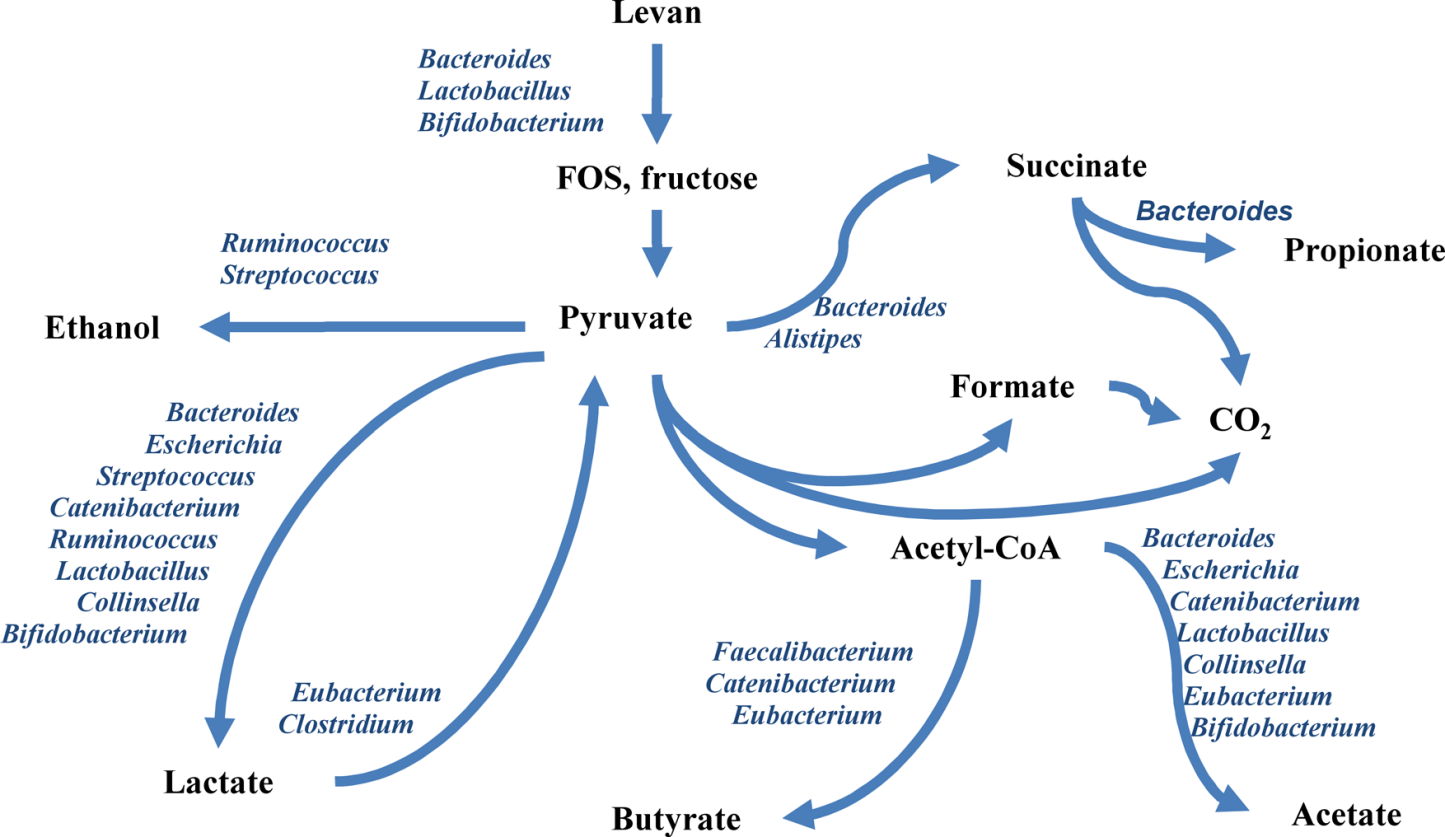
Proposed scheme for levan degradation and product formation on the basis of our experimental data and information from the literature.

The main fermentation products from levan comprised acetic, lactic, butyric, propionic and succinic acids and carbon dioxide. This suggests syntrophic growth of levan-degrading and butyrate producing bacteria. The rate of levan hydrolysis depends on abundance of levan-degrading bacteria in the population. There are only few bacterial genera that harbor strains possessing enzymes for levan degradation. Among the gut bacteria, *B*. *thetaiotaomicron* has the most efficient and best characterized specialized enzymatic system (both endo- and exolevanases) for levan degradation and utilization [16,17]. Some probiotic bacteria such as lactobacilli and bifidobacteria can also catabolize levan. For example *Lactobacillus paracasei* has cell wall bound aspecific exo-acting β-fructosidase that hydrolyzes levan, inulin and various FOS [37,38]. Porras-Domiguez *et al*. [39] reported on growth of *L*. *paracasei* and also of four strains of bifidobacteria on levan and levan-derived FOS. Several *Bacillus* species also possess genes for levan-degradation [40]. The levan-degrading bacteria act in primary step of cascade reactions leading to levan metabolism. The hydrolysis products, FOS and fructose, but also the excreted metabolites such as organic acids and gases can be used by a variety of colon bacteria resulting in specific metabolic patterns and microbiota dynamics. Based on the experimental data, the metabolic flowchart and respective bacteria participating in degradation of levan and production of metabolites was proposed (Fig 4). Importantly, we observed specific growth stimulation of *Faecalibacterium*, *Erysipelotrichaceae* and *Eubacterium* by levan, the growth of these bacteria was not supported in the medium without levan (Fig 1B).

A short first growth phase followed by a second long growth phase may indicate low initial content of levan-degrading bacteria in the fecal sample (Fig 2). This assumption is supported also by the enrichment of specific species from the consortia. Levan is degraded mainly by some *Bacteroides* and *Lactobacillus* species, but following growth of butyrate-producing bacteria (*Faecalibacterium*, *Roseburia*, *Catenibacterium*) and bifidobacteria, streptococci and *Escherichia* indicates that fructose or FOS are efficiently fermented by these bacteria (Fig 1B).

Using a single fermenter system for cultivation experiments with fecal inocula, Duncan *et al*. [41] showed that inulin can affect the growth of different bacterial groups. For example, enrichment of *Bacteroides* strains and disappearance of *Faecalibacterium prausnitzii* was reported. In our study enrichment of taxon *Faecalibacterium* which is also regarded as an abundant representative in healthy gut microbiota, was observed in fecal cultures grown on levan and also on levan + amino acids.

Acidity (pH) has been shown to be a crucial factor that determines the growth and metabolism of colon microbiota [32,33,41]. The main limitation of simple batch cultures such as IMC experiments is that pH cannot be kept within a certain range. Formation of organic acids from the complex carbohydrates leads to the drop of pH that affects enzyme activities of the microbes and results in compositional changes. This may be the main reason of accumulation of lactic acid in the end of growth and inhibition of its further fermentation to other acids. In case of continuous cultures at pH 6.9 of the same fecal inocula, accumulation of lactic acid was not observed (data not shown).

The experiments of Belenguer *et al*. [42] suggest that lactate is rapidly converted to acetate, butyrate and propionate by the human intestinal microbiota at pH values as low as 5.9. However, at pH 5.2 reduced utilization occurs while production is maintained, resulting in lactate accumulation. In addition, 100-fold increase in population of *Eubacterium hallii*, a lactate-utilizing butyrate-producing bacterium was observed at pH 5.9 and 6.4. On the contrary, Walker *et al*. showed that butyrate production by human fecal microbiota was higher at pH 5.5 compared to that at pH 6.5 and it also depended on peptide supply [32]. While the growth of *Bacteroides* was inhibited at lower pH, the isolates of *Roseburia* grew equally well at both pH-s. In fecal samples from healthy donors, lactate has not detected or is present at low concentrations (3 mmol/L) [42], due to its further metabolism in the colon. Since lactate is a major fermentation product of several bacterial families it was proposed that it might contribute to some of the properties attributed to probiotic microorganisms [43]. Still, lactate can modulate inflammatory activation of the epithelial cells. Intraluminal levels of lactate derived from fermentative metabolism of lactobacilli have been shown to modulate inflammatory environment in intestinal mucosa [43].

Along with growing knowledge, the significance of proper sample collection, storage and preparation techniques to obtain adequate results is increasing. Preservation and re-cultivations of live fecal cultures have been applied for FMT (fecal microbiota transplantation) procedures [44,45]. No significant differences were detected between different sampling and storage conditions (freezing vs cooling) on the bacterial community structure at DNA level by Tedjo *et al*. [46]. Composition, growth and function of complex microbial consortia are most often described on the basis of the metagenome or 16S rDNA sequencing data, however, for cultivation studies alive and metabolically active fecal cultures should be used. Thus, the growth experiments are often performed with freshly collected fecal samples and typically not in biological replicates. Resuspension of fecal material in a dialysate solution supplemented with glycerol and stored at -80°C was suggested by Aguirre *et al*. [47] as optimal preservation technique of fecal material for *in vitro* fermentations alternative to using fresh feces. We have shown that fecal slurries in PBS containing 5% DMSO remain metabolically active when stored at -80°C and can be used in repeated cultivation experiments. Chemical composition of the fecal samples during at least 3 months of storage did not change (Table 1). Stability of the frozen feces was also supported by small standard deviations of growth characteristics (Fig 2) calculated from the power time curves (parallel experiments were made within a three month period).

### Proposed use of IMC for gut microbiota studies

Isothermal microcalorimetry (IMC) has been extensively used in studies of pure cultures [17,21,23] and also for mixed cultures of two or three microbial strains [48]. Despite the difficulty in interpretation of the power-time curves of complex microbial consortia due to multiple concurrent metabolic processes, IMC complemented with metagenomic and metabolomic analysis provides the most sensitive approach to monitor the adjustment and development of complex microbial populations under various nutritional conditions. Moreover, significantly higher number (up to 48) of samples and/or environmental conditions can be screened during the same run by using a multichannel calorimeter when compared to conventional fermentation techniques. The latter method is definitely better suited for further detailed metabolic analyses but not for initial screening.

**Fig 5:**
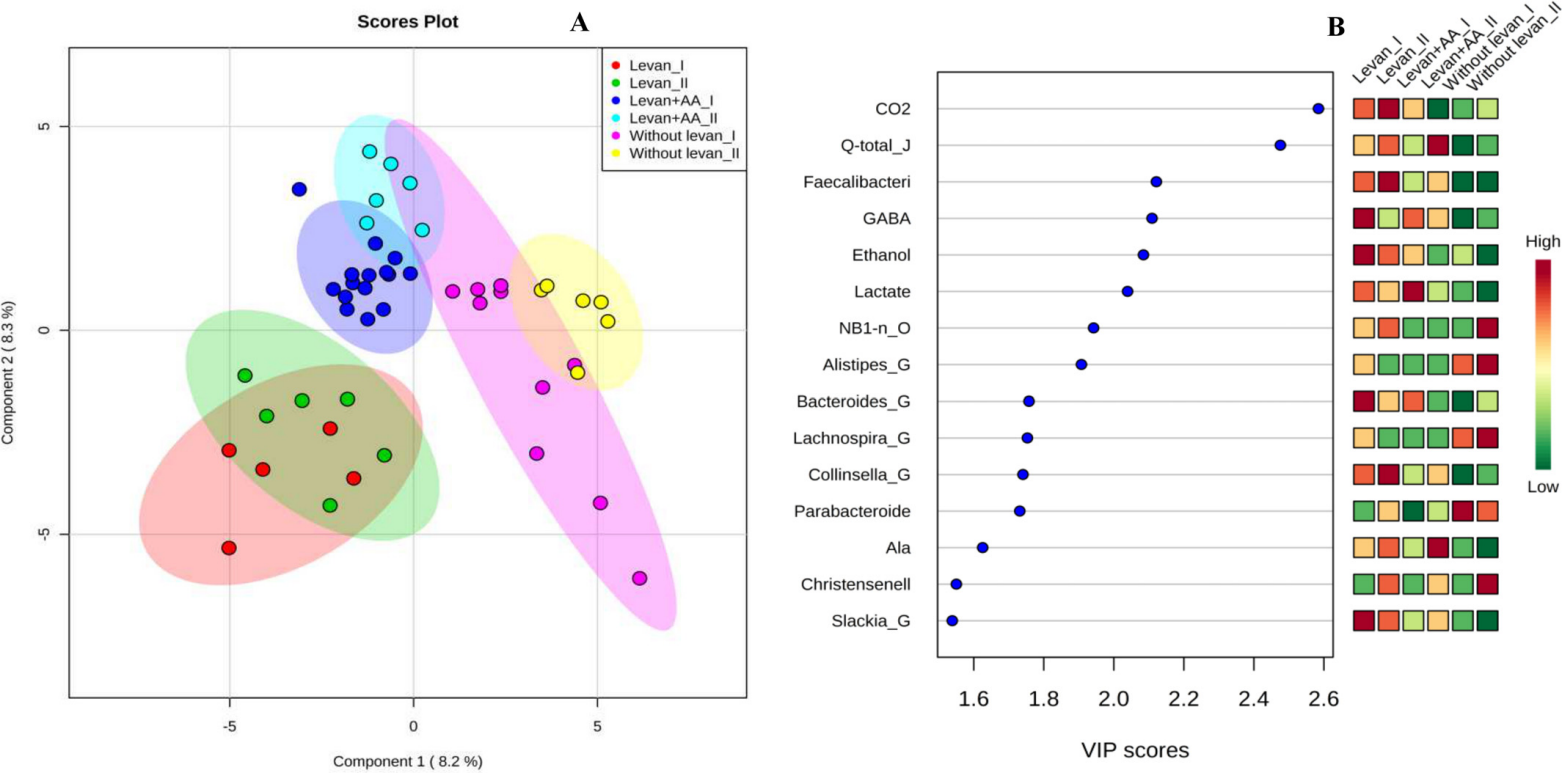
Score plots of PLS-DA (A) and variable importance in projection (VIP) (B). Plots derived from integrated analysis of microcalorimetric data (total heat accumulated (Q), maximal heat evolution rate (P_max_), specific growth rate (μ)), consumption of substrates (levan, amino acids) and formation of products (organic acids, gases and ethanol), and the bacterial genera grown in microcalometry experiments. Analysis was done by MetaboAnalyst 3.0 program [25]. Levan+AA–medium containing levan and 20 amino acids. Groups I and II indicate the fecal samples of FS1-5 and FS6-11, respectively.

A specific advantage of the IMC is applicability of the method to monitor growth in turbid and solid environments (*e*.*g*. fecal slurries). Although direct correlation between the power-time curves with the changes in consortia structure and metabolism may not exist, one can follow the metabolism and dynamics of the colon microbiota on different substrates by combining the growth data with chemical and metagenomics analyses (Fig 5A). Our data show that the growth of complex fecal microbial consortia is determined by multiple factors. The parameters with the highest impact in the case of using levan as the carbon source comprise the heat accumulated, GABA and lactate production, and the growth of *Faecalibacterium* (Fig 5B). Hence IMC is a promising method for comparing fermentation capacity of a persons’ individual gut microbiota on various carbohydrates either singly or in combinations. It can also be applied for screening the day-to-day variation of the fermentation potential of a person, effect of the diet and evaluation of residual energetic values in excreted material.

Compared to the ancient diet, the proportion of dietary fibers in our everyday food has dramatically decreased during the last century. It is generally accepted that restoring the variety and amount of dietary fibers would be the easiest way to enrich and diversify the gut microflora and thereby improve our health. In general, non-digestible oligosaccharides, in particular FOS, are considered as prebiotics [49]. For a long time prebiotic effects have been linked mostly to enhancement of growth of bifidobacteria and lactobacilli. Literature data show that many strains of both these bacteria can grow on levan [39]. From this aspect, levan should certainly be considered as a potential candidate for prebiotic. In the current study we show that levan also enhanced the growth of *Faecalibacterium*. This genus comprises *F*. *prausnitzii*, a butyrate-producing species that is considered a new probiotic bacterium with excellent anti-inflammatory properties [50]. We assume that levan may be suitable as selective substrate for this bacterium.

## Conclusions

The interactions taking part in the gut and their influences on human health are highly complex and we are just in the beginning of understanding the mechanisms. The overall effects of dietary fibers comprise modification of the whole microbial population of the gut by stimulating growth and metabolism of certain groups of bacteria (changing proportions and interactions) and positively influencing metabolic fluxes leading to a diverse range of health benefits.

We showed here that metabolic potential of colon microbiota on specific substrates can be evaluated by isothermal microcalorimetry coupled with product and consortia analysis. Using this approach, the selective effect of known or predicted prebiotic substrates on specific bacteria and/or groups within complex consortia can be revealed. Our experiments clearly indicated a modulating effect of levan on human colon microbiota. The wider influence of this novel candidate prebiotic on human organism should be more thoroughly investigated using *in vitro* as well as *in vivo* methods.

## Supporting Information

S1 Table Raw sequencing data of fecal samples. All samples were normalized to total OTUs of 5971.
(XLSX)

S2 Table Power data (W) of microcalorimetry experiments. (XLSX)

S3 Table Amino acids, biogenic amines and organic acids in the fecal sample aliquotes stored dimethyl sulfoxide (DMSO) and analysed before the calorimetry experiment. Empty cells show the not analysed compounds in the samples.
(XLSX)

S4 Table Metabolite consumptions and productions (mmol/gDW), and grown bacteria (gDW/gDW-total) during the calorimetry experiments. (XLSX)
